# A combination of temsirolimus, an allosteric mTOR inhibitor, with clofarabine as a new therapeutic option for patients with acute myeloid leukemia

**DOI:** 10.18632/oncotarget.762

**Published:** 2012-12-18

**Authors:** Francesca Chiarini, Annalisa Lonetti, Gabriella Teti, Ester Orsini, Daniela Bressanin, Alessandra Cappellini, Francesca Ricci, Pier Luigi Tazzari, Andrea Ognibene, Mirella Falconi, Pasqualepaolo Pagliaro, Ilaria Iacobucci, Giovanni Martinelli, Sergio Amadori, James A. McCubrey, Alberto M. Martelli

**Affiliations:** ^1^ Institute of Molecular Genetics, National Research Council, Bologna, Italy; ^2^ Muscoloskeletal Cell Biology Laboratory, IOR, Bologna, Italy; ^3^ Department of Biomedical and Neuromotor Sciences, University of Bologna, Bologna, Italy; ^4^ Department of Human, Social and Health Sciences, University of Cassino, Cassino, Italy; ^5^ Immunohematology and Transfusion Center, S. Orsola-Malpighi Hospital, Bologna, Italy; ^6^ Department of Specialist, Diagnostic and Experimental Medicine, University of Bologna, Bologna, Italy; ^7^ Department of Hematology, Tor Vergata University Hospital, Rome, Italy; ^8^ Department of Microbiology & Immunology, Brody School of Medicine, East Carolina University, Greenville, NC, USA

**Keywords:** AML, PI3K/Akt/mTOR signaling, apoptosis, autophagy, combination therapy, leukemia initiating cells

## Abstract

Signaling through the phosphatidylinositol 3-kinase (PI3K) pathway and its downstream effectors, Akt and mechanistic target of rapamycin (mTOR), is aberrantly activated in acute myeloid leukemia (AML) patients, where it contributes to leukemic cell proliferation, survival, and drug-resistance. Thus, inhibiting mTOR signaling in AML blasts could enhance their sensitivity to cytotoxic agents. Preclinical data also suggest that allosteric mTOR inhibition with rapamycin impaired leukemia initiating cells (LICs) function. In this study, we assessed the therapeutic potential of a combination consisting of temsirolimus [an allosteric mTOR complex 1 (mTORC1) inhibitor] with clofarabine, a nucleoside analogue with potent inhibitory effects on both ribonucleotide reductase and DNA polymerase. The drug combination (CLO-TOR) displayed synergistic cytotoxic effects against a panel of AML cell lines and primary cells from AML patients. Treatment with CLO-TOR induced a G_0_/G_1_-phase cell cycle arrest, apoptosis, and autophagy. CLO-TOR was pro-apoptotic in an AML patient blast subset (CD34^+^/CD38^−^/CD123^+^), which is enriched in putative leukemia initiating cells (LICs). In summary, the CLO-TOR combination could represent a novel valuable treatment for AML patients, also in light of its efficacy against LICs.

## INTRODUCTION

Acute myeloid leukemia (AML) is defined as a clonal disorder characterized by the uncontrolled proliferation and survival of immature myeloid progenitors that undergo a differentiation block at various maturation steps, leading to accumulation of leukemic cells in bone marrow and inhibition of normal hematopoiesis [1]. Despite advances in the treatment, the cure of patients with AML remains challenging and difficult [2]. In fact, although intensive chemotherapy induces disease remission in a large number of patients, many of them eventually relapse and die. In particular, the outcome of patients aged ≥60 years remains highly disappointing [3]. Elderly patients respond less satisfactory to standard chemotherapy than younger individuals, as reflected by higher incidence of treatment-related mortality, lower complete remission rates, and shorter survival in major clinical trials [4, 5]. Overall, AML prognosis remains dismal and new therapeutic approaches are therefore under active investigation [6]. In AML, aberrant activation of several signal transduction pathways enhances survival, proliferation, and drug-resistance of leukemic cells. Therefore, these signaling cascades are attractive targets for the development of innovative therapeutic strategies for AML patients [7]. One promising target for molecular therapy in AML is the phosphatidylinositol 3-kinase (PI3K)/Akt/mechanistic target of rapamycin (mTOR) signaling pathway, which is constitutively activated in 70-90% of AML patients and has been shown to be central to the proliferation, survival, and drug-resistance of leukemic cells [8, 9]. A major problem in the efforts to treat AML is the inability of current therapies to efficiently target and eliminate leukemia initiating cells (LICs), which are the cells that are thought to initiate and maintain the leukemic phenotype [10]. The majority of LICs are quiescent and therefore not sensitive to various chemotherapeutic agents that kill rapidly dividing cells [11]. This fact could explain the difficulties in eradicating AML with chemotherapy alone and the relapses seen in the majority of patients, despite initial complete responses with drugs regimens [12]. However, mTOR inhibitors, such as rapamycin, have displayed cytotoxic activity against LICs in pre-clinical models of AML [13].

In a recent multicenter, open-label phase II trial (AML1107) performed by the GIMEMA cooperative group, it has been studied the efficacy and safety of the drug combination consisting of low-dosage clofarabine with the allosteric mTOR complex 1 (mTORC1) inhibitor temsirolimus (CCI-779, Torisel^©^) in a group of elderly patients with refractory/relapsed AML [14]. The results from this study documented that temsirolimus could be safely combined with low-dosage clofarabine and that the combination displayed some encouraging clinical activity [14]. Clofarabine is a a second-generation purine nucleoside analogue which has been synthesized to overcome the limitations and incorporate the best properties of fludarabine and cladribine. Clofarabine mainly acts by inhibiting ribonucleotide reductase and DNA polymerase, thereby depleting the amount of intracellular deoxynucleoside triphosphates available for DNA replication [15]. However, clofarabine displayed cytotoxic activity also against non-proliferating leukemia cells, by directly targeting the mitochondria and inducing apoptosis [16].

In the present study, we have investigated the in vitro effects of the clofarabine-temsirolimus (henceforth CLO-TOR) combination on a panel of AML cell lines and primary cells from AML patients. Our results could contribute to design in the future more effective therapeutic protocols based on these two drugs.

## RESULTS

### The CLO-TOR combination has synergistic cytotoxic effects in AML cells

**Figure 1:**
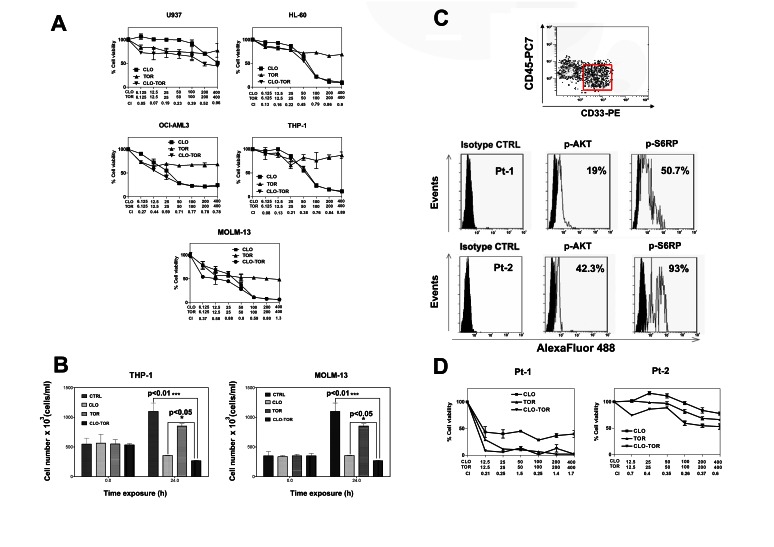
CLO-TOR affects viability of AML cell lines and blasts from AML patients with constitutively active PI3K/Akt/mTOR signaling pathway A: MTT assays performed after 24 h of treatment with the drugs. Results are the mean of at least three different experiments ± s.d. The combination index (CI) value for each data point was calculated with the appropriate software for dose effect analysis (CalcuSyn). B: To assess cell proliferation, 3 × 10^5^ cells were seeded in 25 cm^2^ flasks and growth curves were determined by direct count of cells harvested at 24 h after seeding. Viable cells were counted in a hemocytometer, using 0.2% Trypan Blue. C: Primary cells from AML patients, gated for CD33^+^/CD45^low^ expression (red rectangle), were fixed, permeabilized, stained with AlexaFluor^®^488-conjugated antibodies to either Ser 473 p-Akt, or Ser 235/236 p-S6RP, then analyzed by flow cytometry. Two representative patients are shown. Panel D: MTT assays of AML primary cells treated with clofarabine, temsirolimus, or CLO-TOR for 96 h. Results are the mean of at least three different experiments ± SD. The combination index (CI) value for each data point was calculated as detailed above. In A,B, and D, CTRL, untreated cells; CLO, clofarabine ; TOR, temsirolimus.

The effects of clofarabine, temsirolimus, and their combination (CLO-TOR) on AML cell lines were analyzed by first treating the cells with increasing concentrations of the drugs and then measuring the rates of survival by MTT assays. Cell lines (U937, HL-60, OCI-AML3, THP-1, and MOLM-13) were cultured in the presence of clofarabine or temsirolimus either alone or in combination at a fixed ratio (1:1) for 24 h (Fig. 1A). The combined treatment was highly effective in inducing cytotoxicity in all of the cell lines. The combination index (CI) values, calculated with Calcusyn software for dose-effect analysis, indicated the existence of a strong synergism between clofarabine and temsirolimus (CI<0.3), especially at lower and intermediate drug concentrations. The effects of the drugs on proliferation of THP-1 and MOLM-13 cell lines were analyzed also by cell counting. Exponentially growing cells were seeded and incubated with clofarabine (100 nM), temsirolimus (100 nM), and CLO-TOR (both drugs at 100 nM) for 24 h, then viable cells were counted. Analysis of the results demonstrated that CLO-TOR was more efficacious than either treatment alone in blocking cell proliferation (Fig.1B). To better assess the effectiveness of CLO-TOR as a potential therapeutic combination in AML, we examined the activation of the PI3K/Akt/mTOR pathway in 12 AML patient samples isolated from bone marrow or peripheral blood. Pathway activation was studied by flow cytometry, after staining the samples with a phycoerythrin (PE)-conjugated anti-CD33 and a PC7-conjugated anti-CD45 antibody, followed by intracellular staining for either Ser 473 p-Akt or Ser 235/236 p-S6 ribosomal protein (S6RP). Therefore, blast cells could be positively identified by CD33/CD45/side scatter gating (Fig.1C). All of the analyzed samples displayed activation of the signaling pathway, as documented by phosphorylation of both Akt and S6RP (Fig.1 C).

AML primary cells were then treated with the drugs, and cell viability was analyzed by MTT assays. A marked reduction in cell viability at 96 h was detected (Fig.1D). In Fig.1D it is shown a patient who was very sensible to CLO-TOR and a patient more resistant to the treatment, but in whom nevertheless the CLO-TOR combination displayed a strong synergism, with CIs < 0.4. Of the 12 analyzed patients, 8 were sensitive whereas 4 displayed various degree of resistance to the drug combination. Overall, these findings demonstrated that the CLO-TOR combination has a synergistic cytotoxic activity also against primary cells from AML patients with up-regulated PI3K/Akt/mTOR signaling.

### The CLO-TOR combination has pro-apoptotic effects on AML cell lines and block cells in the G_0_/G_1_ phase of the cell cycle

**Figure 2:**
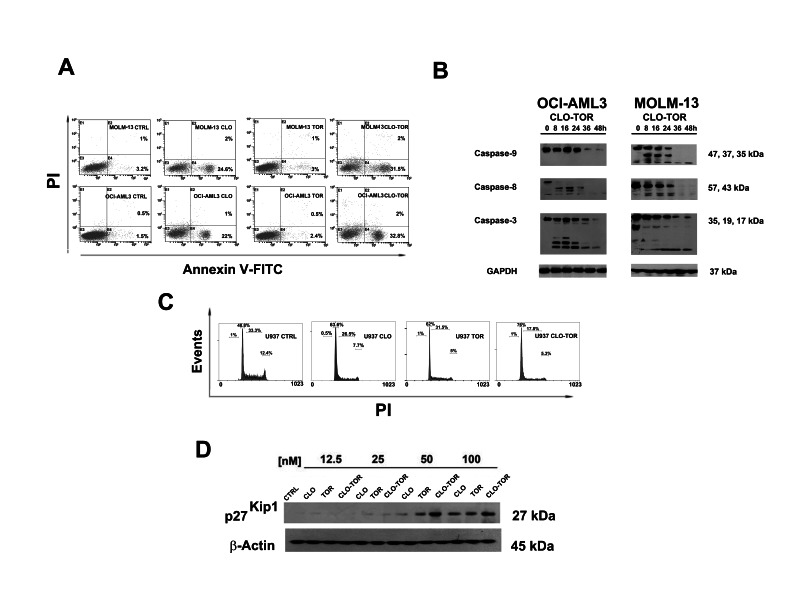
CLO-TOR induces apoptosis and cell cycle arrest in the G_0_/G_1_ phase of cell cycle A: Flow cytometric analysis of Annexin V-FITC/PI stained AML cell lines treated for 8 h with 100 nM clofarabine (CLO), 100 nM temsirolimus (TOR), or the CLO-TOR combination (100 nM + 100 nM). The percentages of early apoptotic cells (Annexin-V FITC^+^/PI^−^, lower right quadrant) and late apoptotic/necrotic cells (Annexin-V FITC^+^/PI^+^, upper right quadrant) are indicated. The histograms are representative of three separate experiments. CTRL: untreated cells. B: Western blot analysis documenting the activation of caspase-8, caspase-9, and -3 by CLO-TOR (100 nM + 100 nM). Cells were treated with CLO-TOR for the indicated times, collected, and then lysed. Fifty micrograms of each lysate were electrophoresed on SDS-PAGE gels, followed by transfer to nitrocellulose membranes. An antibody to GAPDH demonstrated equal lane loading. Molecular weights are indicated at right. C: Flow cytometric analysis of PI-stained U937 cells after 24 h of treatment with clofarabine (CLO, 100 nM), temsirolimus (TOR, 100 nM), or the CLO-TOR combination (100 nM + 100 nM). One representative of three separate experiments is shown. D: Western blot analysis documenting concentration-dependent, increased expression of p27^Kip1^ in U937 cells treated with the drugs for 24 h. An antibody to β-actin demonstrated equal lane loading. Molecular weights are indicated at right. CTRL, untreated cells; CLO, clofarabine; TOR, temsirolimus.

It was then investigated whether cell viability impairment could be related to apoptosis, using Annexin V-fluorescein isothiocyanate (FITC)/propidium iodide (PI) staining in AML cell lines. After 8 h of treatment, flow cytometric analysis documented that CLO-TOR was more effective than either treatment alone in inducing apoptosis (Fig. 2A). Western blotting analysis of protein extracts from AML cell lines treated with CLO-TOR documented the cleavage of procaspase-8, -9 and -3 (Fig. 2B).

Given the fundamental role played by PI3K/Akt/mTOR signaling in the control of cell proliferation, the effects of CLO-TOR on cell cycle progression were also investigated. Flow cytometric analysis of PI-stained U937 AML cells treated for 24 h with clofarabine or temsirolimus either alone or in combination documented a marked increase in G_0_/G_1_ phase cells with a decrease in S and G_2_/M phase cells. Again, the combined treatment was more effective than either treatment alone (Fig. 2C). Cell cycle arrest in G_0_/G_1_ was accompanied by an increase in the expression of p27^Kip1^, a negative regulator of cell cycle progression whose levels are under the control of mTOR signaling [17]. However, the CLO-TOR combination was more effective than temsirolimus alone in inducing p27^Kip1^ expression and this was consistent with the effects on cell cycle progression.

### The CLO-TOR combination induces autophagy

**Figure 3:**
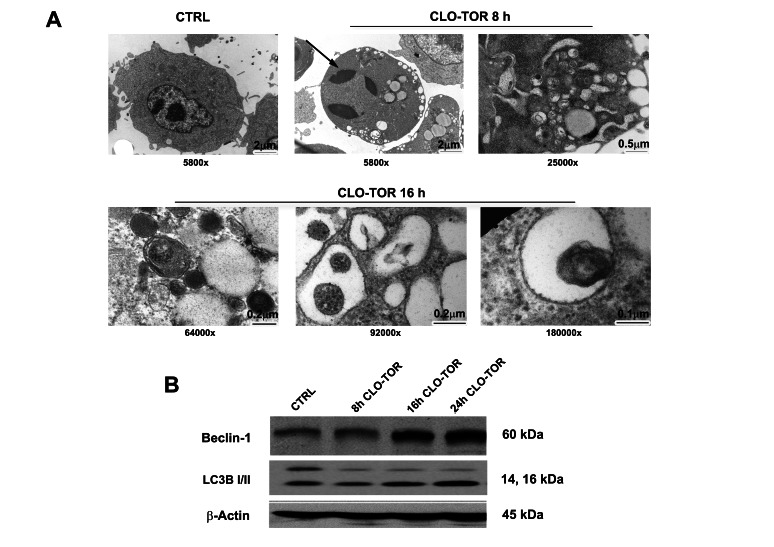
CLO-TOR induces autophagy in MOLM-13 cells A: Cells were treated for the indicated times with CLO-TOR (100 nM + 100 nM), then processed for TEM analysis that documented both autophagy and apoptosis induction. Large cytoplasmic vacuoles containing various degraded organelles are evident, as well as condensed apoptotic chromatin (arrow). The original magnification of the micrographs is indicated. B: Western blot analysis for Beclin-1 and LC3B I/II expression in MOLM-13 cells treated with CLO-TOR (100 nM + 100 nM) for the indicated times. An antibody to β-actin demonstrated equal lane loading. Molecular weights are indicated at right. In A and B, CTRL, untreated cells.

As mTORC1 is an inhibitor of autophagy, it was investigated whether the CLO-TOR combination could induce autophagy in AML cells. Induction of autophagy was confirmed by transmission electron microscopy (TEM) analysis, which documented the presence of autophagic vacuoles in the cytoplasm of MOLM-13 cells (Fig. 3A). However, TEM analysis after CLO-TOR treatment also revealed the presence of apoptotic cells and, intriguingly, we found both apoptotic and autophagic features in the same cells (Fig.3A). To confirm induction of autophagy, we studied the expression levels of both beclin-1 and LC3B I/II, two recognized autophagy markers [18]. Western blot analysis demonstrated an increase in the expression of both beclin-1 and of the fast migrating form of LC3B (LC3B II, 14 kDa), while LCB I (16 kDa) decreased (Fig. 3B). It is worth remembering here that LC3B-II is bound to the membrane of autophagosome [19].

### The CLO-TOR combination affects PI3K/Akt/mTOR signaling and p-ERK levels in AML cell lines

**Figure 4:**
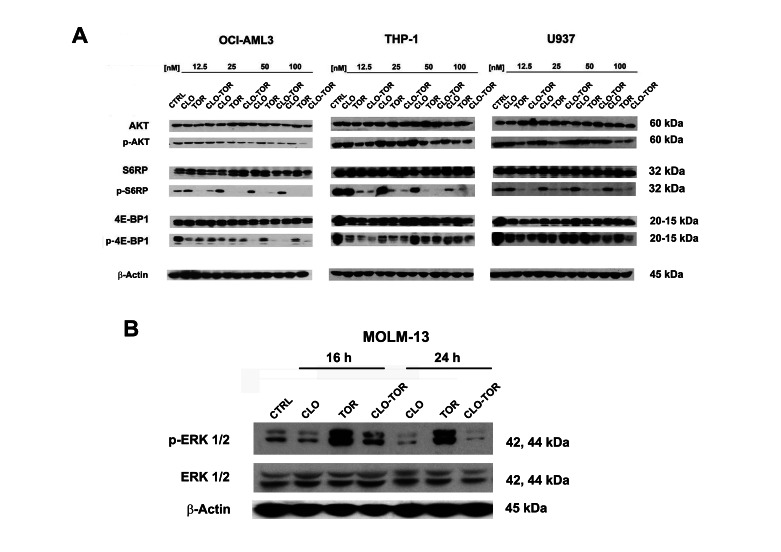
CLO-TOR affects the phosphorylation state of critical components of the PI3K/Akt/mTOR signaling pathway, as well as p-ERK 1/2 levels in AML cell lines A: Cells were treated with clofarabine (CLO), temsirolimus (TOR), or the drug combination (CLO-TOR) at the indicated concentrations for 24 h, collected, and lysed. Western blot analysis was then performed. B: Western blot analysis documenting changes in p-ERK 1/2 levels in MOLM-13 cells treated with the single drugs (100 nM) or CLO-TOR (100 nM + 100 nM) for the indicated times. In A and B, 50 μg of each lysate were electrophoresed on SDS-PAGE gels, then blotted to nitrocellulose membranes. An antibody to β-actin demonstrated equal lane loading. Molecular weights are indicated at right. In A and B, CTRL, untreated cells.

We studied the effects of increasing concentrations of the drugs administered alone or in combination for 24 h on components of the PI3K/Akt/mTOR signaling pathway. Western blotting analysis documented that dephosphorylation of various key components of the signaling network (Akt, S6RP, 4E-BP1) was mainly due to the effect of either temsirolimus or CLO-TOR combination, while clofarabine alone was not able to induce a significant modulation of this signaling cascade (Fig.4A). Overall, CLO-TOR was more powerful than temsirolimus alone in inducing protein dephosphorylation. The decrease in the levels of Ser 473 p-Akt was consistent with the capacity of CLO-TOR combination to inhibit also mTORC2, which is involved in Ser 473 p-Akt phosphorylation [20].

Considering the relevance of ERK signaling in AML cells [21, 22], it was analyzed the phosphorylation levels of ERK 1/2 in MOLM-13 cells treated with the drugs. Western blotting analysis documented that clofarabine slightly decreased p-ERK 1/2 levels, whereas temsirolimus dramatically increased them. However, the CLO-TOR combination completely prevented the increase in p-ERK 1/2 induced by temsirolimus (Fig. 4B).

### The CLO-TOR combination modulates STAT3 and c-Myc in AML cells

**Figure 5:**
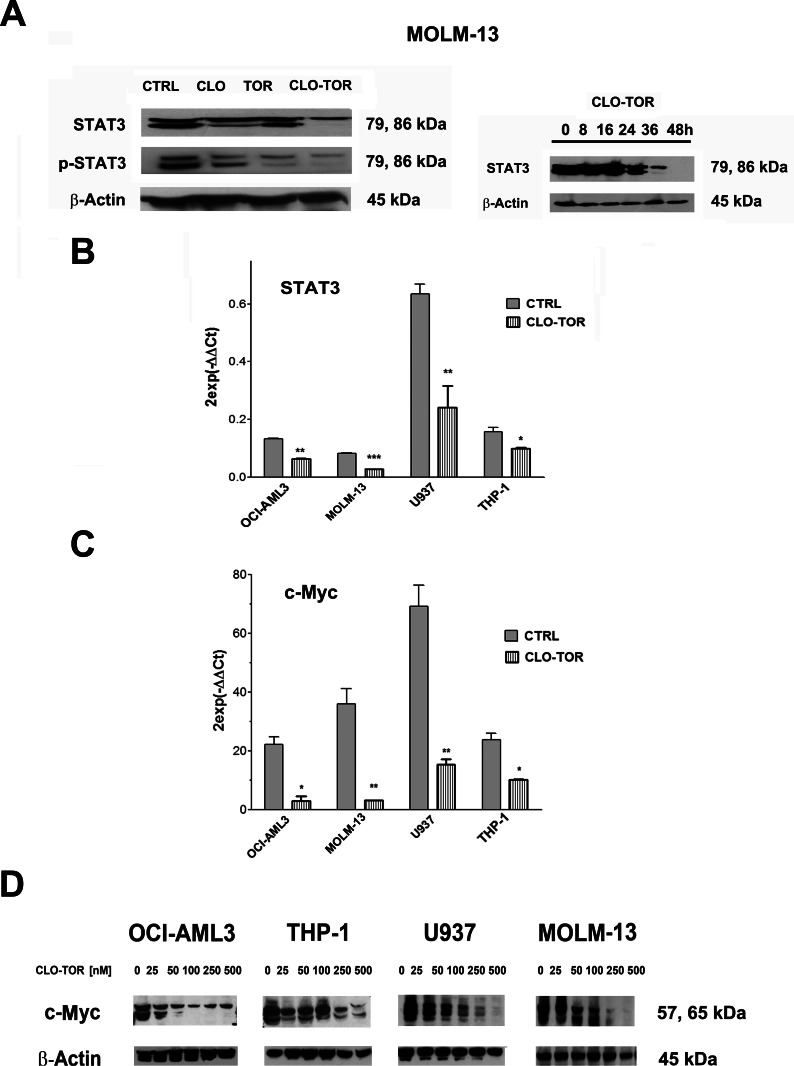
CLO-TOR modulates STAT3 and c-Myc expression in AML cell lines A: Western bot analysis for STAT-3 and Ser 727 p-STAT3 in MOLM-13 cells treated with clofarabine (CLO, 100 nM), temsirolimus (TOR, 100 nM), or CLO-TOR (100 nM + 100 nM) for 48 h. B: Western blot analysis documenting a time-dependent reduction in STAT3 expression levels in MOLM-13 cells treated with CLO-TOR (100 nM + 100 nM). In A and B, an antibody to β-actin demonstrated equal lane loading. Molecular weights are indicated at right. B: qRT-PCR for STAT3 mRNA expression in AML cell lines treated with CLO-TOR (100 nM + 100 nM) for 24 h. Results are the mean from three different experiments ± s.d. C: qRT-PCR for c-Myc mRNA expression in AML cell lines treated with CLO-TOR (100 nM + 100 nM) for 24 h. Results are the mean from 3 different experiments ± s.d. In A-C, CTRL, untreated cells. D: Western bot analysis for c-Myc protein expression in AML cell lines treated with different concentration of CLO-TOR for 48 h. An antibody to β-actin documented equal lane loading. Molecular weights are indicated at right.

It is well known that up-regulated STAT3 activity is associated with several human tumors, including AML [23] and that STAT3 inhibition can mediate tumor regression [24]. Recently, it has been documented that mTORC2 controls phosphorylation of STAT3 at Ser 727 [25]. Therefore, we analyzed Ser 727 p-STAT3 levels in MOLM-13 cells treated with the drugs. Western blot analysis demonstrated that STAT3 was dephosphorylated at Ser 727 maximally after treatment with CLO-TOR for 48 h (Fig. 5A). However, we also observed a reduction in the amount of total STAT3. Quantitative real-time PCR (qRT-PCR) analysis documented that this reduction was likely due to decreased expression of the STAT3 gene, that was detected in all AML cell lines already after 24 h of treatment with CLO-TOR (Fig. 5B). Similar results were detected in AML primary cells (data not shown).

Phosphorylation of STAT3 at Ser 727 is necessary, in addition to tyrosine phosphorylation, for full activation of STAT3-dependent transcription [26]. Known STAT3 up-regulated genes include Bcl-xL, Mcl-1, survivin, Akt, and c-Myc [27]. Therefore, we analyzed the levels of c-Myc by both qRT-PCR and western blotting that documented a marked decrease in the expression of c-Myc mRNA and protein in all AML cell lines we studied (Fig. 5C-D).

### Effects of CLO-TOR on mTORC1 signaling and p-ERK 1/2 in AML primary cells

**Figure 6:**
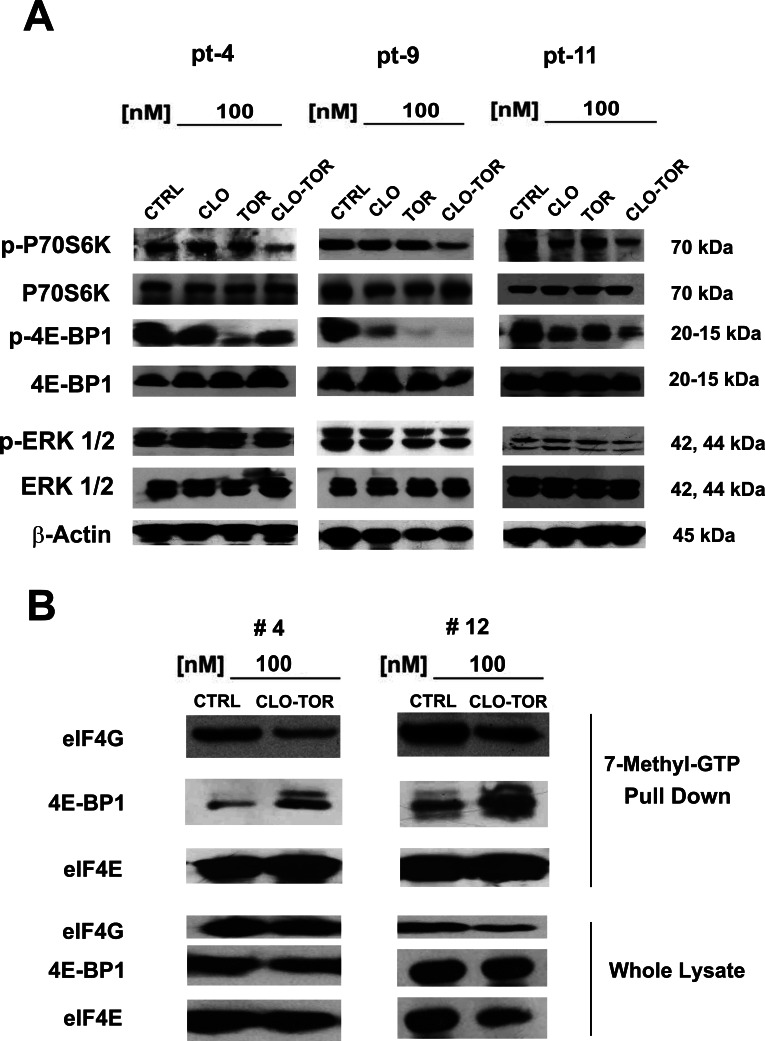
CLO-TOR affects mTORC1 signaling and eIF4F complex formation in AML patient samples A: AML patient samples were cultured for 24 h in the presence of clofarabine (CLO, 100 nM), temsirolimus (TOR, 100 nM), or CLO-TOR (100 nM + 100 nM). Cell lysates were clarified by centrifugation, separated by SDS-PAGE and blotted to nitrocellulose membranes that were incubated with antibodies to Thr 389 p-p70S6K, p70S6K, Thr 37/46 p-4E-BP1, 4E-BP1, Thr 202/Tyr 204 p-ERK 1/2, and ERK 1/2. An antibody to β-actin documented equal lane loading. Molecular weights are indicated at right. Three representative samples are shown. CTRL, untreated cells. B: AML patient primary cells treated with CLO-TOR (100 nM) were lysed in solubilizing buffer. Lysates were clarified by centrifugation and incubated with 7-methyl-GTP-Sepharose beads in binding buffer. Beads were then washed three times in binding buffer and boiled in Laemmli's sample buffer. Protein levels were then analyzed by western blot. Two representative patients are shown. CTRL, untreated cells.

We analyzed the effect of CLO-TOR on mTORC1 signaling and p-ERK 1/2 in 12 AML patient samples. Three representative patients are presented in Fig. 6A. Overall, the CLO-TOR combination was more powerful than either drug alone in dephosphorylating both p70S6 kinase (p70S6K) and 4E-BP1. However, in contrast to the results obtained using MOLM-13 cells, the drugs did not result in either increased or decreased phosphorylation of ERK 1/2 in AML primary cells.

### eIF4F complex formation is down-regulated by CLO-TOR treatment in AML patient samples

Translation of mRNA is tightly regulated at the initiation level through the assembly of eIF4F complexes. The phosphorylation of 4E-BP1 is the limiting step in the assembly of the translation initiating complex eIF4F, initiated by the interactions between the eIF4E and eIF4G proteins [28, 29]. It has been documented that rapamycin and rapalogs were not effective at blocking the formation of the eIF4F complex in acute leukemia cells, owing to incomplete dephosphorylation of 4E-BP1 [30, 31]. We thus performed 7-methyl-GTP pull-down assays in patient samples treated with CLO-TOR to study the interactions between eIF4E and eIF4G (active translation) or 4E-BP1 (inactive translation). CLO-TOR decreased the amount of eIF4G associated with eIF4E, whereas the amount of 4E-BP1 associated with eIF4E increased, thus switching translation to an inactive state (Fig. 6B). Western blots analysis performed on whole-cell lysates documented that the drug combination did not significantly reduce eIF4G, eIF4E, or 4E-BP1 expression.

### The CLO-TOR combination targets the CD34^+^/CD38^−^/CD123^+^ AML cell subset

**Figure 7:**
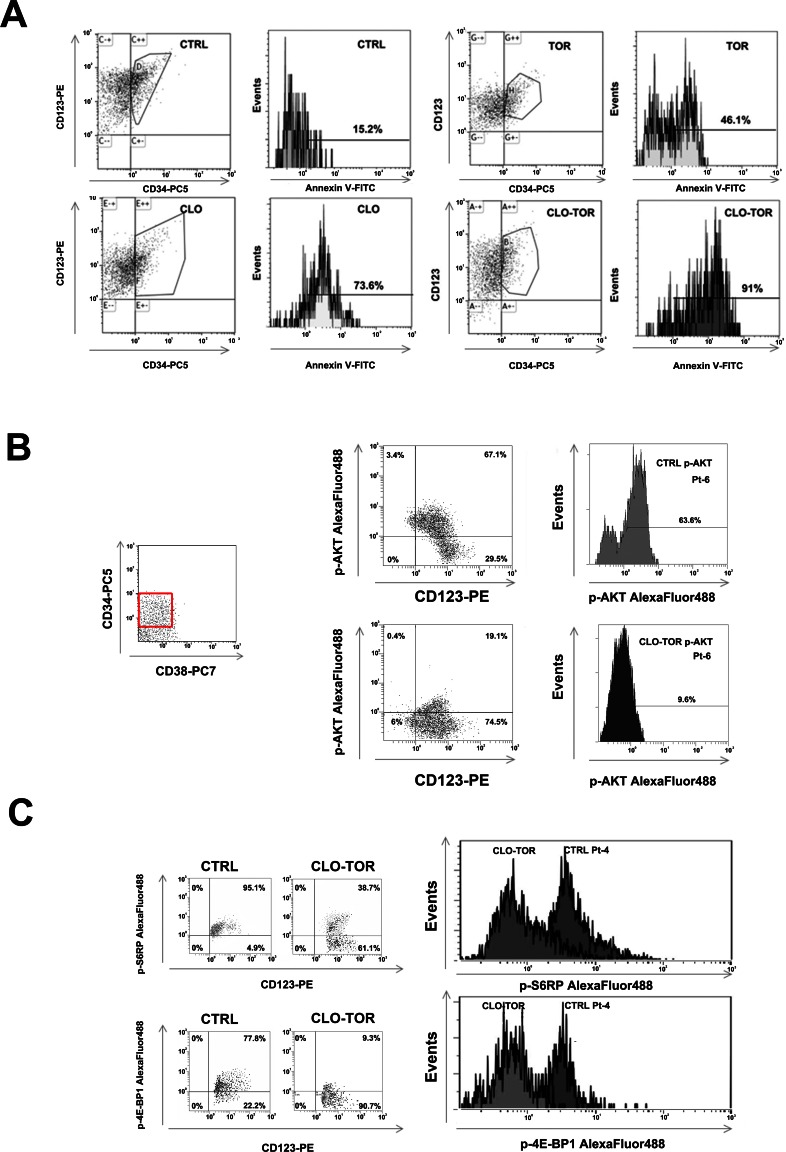
CLO-TOR induces apoptosis and modulates PI3K/Akt/mTOR signaling in the CD34^+^/CD38^−^/CD123^+^ AML patient subpopulation A: Primary cells from AML patients were gated for CD34/CD38/CD123 expression, then the CD34^+^/CD38^−^/CD123^+^ cell subset was analyzed for positivity to Annexin V by flow cytometry, after 24 h of treatment with clofarabine (100 nM, CLO), temsirolimus (100 nM, TOR), or CLO-TOR combination (100 nM + 100 nM). One representative patient is shown. CTRL, untreated cells. B: Primary AML cells were incubated with CLO-TOR (100 nM + 100 nM) for 24 h. After electronic gating on the CD34^+^/CD38^−^ cell subset, CD123^+^ positive cells were analyzed for Ser 473 p-Akt expression levels by flow cytometry. C: As in B, with the exception that putative AML LICs cells were analyzed for Ser 235/236 p-S6RP. In B and C one representative patient is shown. CTRL, untreated cells.

Relapses from AML are thought to originate from the outgrowth of a leukemic subpopulation (CD34^+^/CD38^−^/CD123^+^) displaying both self-renewal and drug-resistance, referred to as LICs. Therefore, it was investigated whether CLO-TOR could induce apoptosis this AML patient cell subset. After electronic gating on the CD34^+^/CD38^−^/CD123^+^ subpopulation, cells were analyzed for positivity to Annexin V staining. After 24 h of treatment, the CLO-TOR combination was significantly more effective in inducing apoptosis than the single drugs (Fig. 7A). The CLO-TOR combination was also able to induce a marked dephosphorylation of both Ser 473 p-Akt (Fig. 7B) and Ser 235/236 p-S6RP (Fig. 7C) in the CD34^+^/CD38^−^/CD123^+^ cell subset, implying a down-modulation of mTORC1/2 signaling activity in LICs.

## DISCUSSION

Chemotherapy is at present the treatment of choice for treating AML patients [32]. However, great interest surrounds the development of novel, lees toxic, targeted therapies, especially for elderly patients [33, 34]. Here, we have evaluated the in vitro effects of the CLO-TOR combination on AML cell lines and patient samples. CLO-TOR reduced cell viability in a panel of AML cell lines and primary cells from AML patients. The combined treatment increased the percentage of cells in the G_0_/G_1_ phase of the cell cycle. Inhibition of cell proliferation correlated with up-regulation of the CDK inhibitor p27^Kip1^. It is known that mTOR controls the expression of the p27^Kip1^ gene through the Oct 1 transcription factor [35]. Nevertheless, we do not know why clofarabine, when administered with temsirolimus, potentiated the induction of p27^Kip1^ expression.

Moreover, the CLO-TOR combination induced apoptosis, as documented by cleavage of caspase-3, -8, and .9. These findings indicated that both the intrinsic and extrinsic pathway of apoptosis were activated upon incubation with the drugs. However, we observed that also autophagy was activated in response to CLO-TOR. It should be reminded here that mTORC1 inhibits autophagy [36]. Interestingly, while clofarabine alone predominantly induced apoptosis (data not shown), the CLO-TOR treatment induced both apoptosis and autophagy in the same cell. Since autophagy could be a protective mechanism against the cytotoxic effects of chemotherapeutic drugs [37], it will be interesting in the future to assess if inhibition of autophagy could increase the cytotoxicity of the CLO-TOR combination.

CLO-TOR was capable of inhibiting the phosphorylation of Akt on Ser 473 as well as phosphorylation of the mTORC1 downstream targets, p70S6K, S6RP, and 4E-BP1 in the cell lines and patient samples analyzed. Down-regulation of Ser 473 p-Akt levels demonstrated that the drug combination was able to inhibit also mTORC2 activity. This observation is noteworthy, as previous studies have highlighted that a major limitation of rapamycin and its analogs is that they could actually up-regulate p-Akt levels in AML cells [38]. The ability of CLO-TOR to down-modulate phosphorylation of 4E-BP1 is also important, as Tamburini et al. [30] emphasized that the weak anti-leukemic activity of the rapalogs was mainly due to the sustained high level of 4E-BP1 phosphorylation in AML cells treated with these compounds. Conversely, we showed herein that the CLO-TOR combination blocked 4E-BP1 phosphorylation at Thr 37/46 in AML patient samples. Indeed, phosphorylation of 4E-BP1 is the limiting step for the assembly of the translation-initiating complexes. As a consequence of 4E-BP1 dephosphorylation, CLO-TOR inhibited the assembly of eIF4F-initiating complexes, as we have demonstrated. This could result in a global inhibition of protein synthesis and thus a decreased expression of oncogenic proteins regulated at the translation initiation level [39].

Another limitation of rapalogs is that mTORC1 inhibition led to the activation of ERK 1/2 in a variety of cancer cell lines and primary tumor cells, as well as in a cohort of patients with metastatic disease who had undergone therapy with RAD-001 [40]. Mechanistically, it has been demonstrated that ERK 1/2 activation could be mediated by a p70S6K-PI3K-Ras signaling pathway [40]. However, the impact of rapalogs on ERK 1/2 phosphorylation levels has never been studied in AML cells. Temsirolimus dramatically increased ERK 1/2 phosphorylation in MOLM-13 cells and, intriguingly, this increase was completely blocked by co-treatment with clofarabine. Nevertheless, we have not detected any changes in p-ERK 1/2 levels in AML primary cells treated with temsirolimus. Therefore, the effects of rapalogs on ERK 1/2 signaling in AML cells is an issue that will require further investigation, using a panel of AML cell lines and a larger number of patient samples.

Another interesting observation emerging from our study is that CLO-TOR treatment affected the transcription of the STAT3 gene in AML cell lines and patient samples. It has been documented that STAT3 gene expression is regulated by mTOR signaling in cancer cells [41, 42], however, it is not clear why the addition of clofarabine to temsirolimus resulted in further down-regulation of STAT3 gene.

Consistently with the down-regulation of STAT3 expression and phosphorylation, CLO-TOR caused a decreased expression of c-Myc gene and protein levels. c-Myc expression down-regulation may be very important for explaining the cytotoxic effects of CLO-TOR, as a decrease in c-Myc levels could result in the inhibition of ribosome synthesis that in turn causes proliferative arrest or have irreversible consequences, including apoptosis [43].

We have also documented that CLO-TOR treatment caused apoptosis in the CD34^+^/CD38^−^/CD123^+^ cell subset that is enriched in LICs. Importantly, the combined treatment was more effective than either drug alone in inducing apoptotic cell death in this subpopulation. Moreover, CLO-TOR was able to down-regulate the phosphorylation of Akt at Ser 473 and of S6RP at Ser 235/236, implying targeting of both mTORC1 and mTORC2 in the CD34^+^/CD38^−^/CD 123^+^ subset.

Although clofarabine is used for the treatment of acute leukemias both in the adults and children [44, 45], surprisingly there are very few data in the literature regarding the effects of this drug on signaling pathways that are up-regulated in leukemic cells. It has been documented that clofarabine induced dephosphorylation of Akt and some of its down-stream targets (Bad and FOXO3A) in CCRF-CEM acute lymphoblastic leukemia cells [46]. Moreover, clofarabine decreased the expression of anti-apoptotic molecules, including Mcl-1 and Bcl-X_L_. Here, we have demonstrated that clofarabine potentiated the effects of temsirolimus on p27^Kip1^ and STAT3 expression, as well as on the phoshorylation levels of Akt, p70S6K, S6RP, and 4E-BP1. It is at present unclear how clofarabine could influence gene expression and protein phosphorylation. However, further studies of these additional effects of clofarabine will be of the utmost importance, as they could help in designing more efficacious therapeutic protocols, combining clofarabine and signal trasduction modulators, for the treatment of acute leukemia patients.

## MATERIALS AND METHODS

### Materials

Clofarabine was provided by Genzyme Europe (Naarden, The Netherlands), while temsirolimus (CCI-779, Torisel^®^) was from Pfizer Inc. (New York, NY, USA). For western blotting analysis, primary antibodies were purchased from Cell Signaling Technology (Danvers, MA, USA). All the antibodies for flow cytometry were from Beckman Coulter (Miami, FL, USA).

### Cell culture and primary samples

The AML cell lines were grown in RPMI 1640, supplemented with 10% fetal bovine serum (FBS). All cell lines, except U937, were from Deutsche Sammlung von Mikroorganismen und Zellkulturen GmbH (Braunschweig, Germany), and were characterized as specified (http://www.dsmz.de/human_and_animal_cell_lines/main.php?contentleft_id=21). Peripheral blood or bone marrow primary cells from AML patients were obtained with informed consent according to Institutional guidelines, isolated using Ficoll-Paque (GE Healthcare, Little Chalfont, UK), and cultured in RPMI 1640 with 20% FBS.

### Cell viability analysis

MTT (3-[4,5-Dimethylthythiazol-2-yl]-2,5-Diphenyltetrazolium Bromide) assays were performed to assess the sensitivity of cells to drugs, as previously described [47, 48].

### Combined drug effect analysis

The combination effect and a potential synergy were evaluated from quantitative analysis of dose-effect relationships, as described elsewhere [49]. For each combination experiment, a CI (combination index) number was calculated using the Biosoft CalcuSyn software. This method of analysis generally defines CI values of 0.9 to 1.1 as additive, 0.3 to 0.9 as synergistic, and <0.3 as strongly synergistic, whereas values >1.1 are considered antagonistic.

### Cell cycle analysis

Flow cytometric analysis was performed using a PI/RNase A staining according to standard procedures, as described previously [50].

### Annexin V-FITC/PI staining

To determine the extent of apoptosis induction, flow cytometric analysis of Annexin V-FITC/PI-stained samples was performed [20]. Samples were analyzed on an FC500 flow cytometer (Beckman Coulter) with the appropriate software (CXP, Beckman Coulter).

### Flow cytometric detection of AML blasts and LICs

Bone marrow mononuclear cells from AML patients were separated by Ficoll/Hypaque density centrifugation and stained with a PE-conjugated anti-CD33 and a PC7-conjugated anti-CD45 antibody. They were then washed with phosphate-buffered saline and processed for intracytoplasmic staining of either p-Akt or p-S6RP, using AlexaFluor488^®^-conjugated antibodies, as reported elsewhere [14].

For LICs detection, a total of 5×10^5^ primary AML blast cells were stained with the following conjugated antibodies: CD34-PC5, CD38-PC7, and CD123-PE. In some cases cells were further stained with Annexin V-FITC, while in others they were permeabilized and stained with AlexaFluor^®^ 488-conjugated antibodies to either p-Akt or p-S6RP [51].

Analyses were performed on a Navios (Beckman Coulter) flow cytometer equipped with Kaluza software (Beckman Coulter). Isotypic controls of the corresponding fluorochromes were used to define the threshold for positive-staining cells.

### Western blotting analysis

This was performed by standard methods, as previously reported [52]. Analysis with an antibody to either GAPDH or β-actin documented equal protein loading.

### TEM analysis

This was performed according to standard techniques [53], using a Philips CM10 (Philips, Eindhoven, The Netherlands) transmission electron microscope. Images were recorded on a Megaview III digital camera (Olympus, Tokyo, Japan).

### 7-Methyl-GTP cap affinity assay

A total of 5 ×10^6^ AML cells were lysed by 3 freeze-thaw cycles in 600 μL of solubilization buffer [20 mM N-2-hydroxyethylpiperazine-N’-2-ethanesulfonic acid/KOH, pH 7.6, 200 mM KCl, 0.5 mM ethylenediaminetetraacetic acid (EDTA), 20 mM KF, 1 mM K_4_P_2_O_7_, 10% glycerol, 1% NP40, protease inhibitors and 50 μ;g/mL RNAse-A]. Cell lysates were clarified by centrifugation (13000 rpm, 20 min, 4°C), and supernatants were incubated (2 h, 4°C) with 7-Methyl-GTP-Sepharose beads (GE Healthcare) in 400 μL binding buffer (50 mM Tris/HCl, pH 7.5, 300 mM KCl, 1 mM EDTA, 20 mM KF, 1 mM K_4_P_2_O_7_, 1 mM dithiothreitol, and protease inhibitors). Beads were then washed 3 times in binding buffer and boiled in Laemmli's sample buffer [30].

### STAT3 and c-Myc expression analysis by qRT-PCR

Total RNA was extracted using the RNeasy Mini Kit (Qiagen, Venlo, The Netherlands) according to the manufacturer's instructions and 1 μg of total RNA was reverse transcribed using Moloney Murine Leukemia Virus Reverse Transcriptase (M-MLV RT) and Oligo(dT) 15 primer in the presence of dNTPs (Promega, Fitchburg, WI, USA). STAT3 and c-Myc gene expression was assessed using The TaqMan® Gene Expression Master Mix, the assays Hs00374280_m1 and Hs00905030_m1 respectively, and the 7300 real-time PCR system (Applied Biosystems, Foster City, CA, USA). Results were normalized to the level of the ubiquitously expressed RNA 18S ribosomal 1 gene (RN18S, Hs03928990_g1) and were expressed as 2^−ΔΔ^Ct.

### Statistical evaluation

The data are presented as mean values from three separate experiments ± s.d. Data were statistically analyzed by a Dunnet test after one-way analysis of variance (ANOVA) at a level of significance of p < 0.05 vs. control samples.
